# Hypertension Characteristics and Neuroimaging Markers of Cerebrovascular Injury in Young Hypertensive Adults With Acute Cerebrovascular Events

**DOI:** 10.1155/srat/4927580

**Published:** 2026-09-15

**Authors:** Mundih Noelar Njohjam, Tiffany Falonne Niakam, Mark Olivier Ngoule

**Affiliations:** ^1^ National University Teaching Hospital, Dakar, Senegal; ^2^ University Teaching Hospital, Montreal, Canada, kreativ.ca

**Keywords:** cerebrovascular injury, hypertension, small vessel disease, stroke, young adults

## Abstract

Stroke among young adults is a growing public health problem in sub‐Saharan Africa, where hypertension is the leading modifiable risk factor for cerebrovascular injury (CVI). We aimed to describe hypertension patterns and associated CVI among young adults with stroke in Senegal. We conducted a cross‐sectional study among adults aged ≤ 50 years with neuroimaging‐confirmed stroke in Senegal. We collected demographic characteristics, vascular risk factors, blood pressure parameters, MRI findings, and clinical outcomes. Hypertension was categorized as previously diagnosed, newly diagnosed, treated, or untreated. We performed multivariable analyses to identify predictors of stroke severity, CVI, and outcomes. A total of 94 patients were included, with a mean age of 45.0 ± 4.3 years; 55.3% were female. Mean admission systolic and diastolic blood pressures were 150.31 ± 31.8 mmHg and 90.83 ± 22.03 mmHg, respectively, and 42.5% presented with severe hypertension. Primary hypertension accounted for 97.9% of hypertension cases. White matter hyperintensities were the most frequent marker of CVI and were significantly associated with stroke severity (*p* = 0.016) and poorer functional outcomes at discharge (*p* = 0.035). In multivariable regression, CVI remained significantly associated with stroke severity (*B* = 3.35, 95% CI 0.18–6.49, *p* = 0.032). Hemorrhagic stroke was associated with higher admission systolic and diastolic blood pressure (*p* = 0.001 for both). Young adults in this cohort demonstrated a high burden of hypertension‐related CVI.

## 1. Introduction

Globally, hypertension is recognized as the most common modifiable risk factor for cerebrovascular diseases [1–3]. Persistent high blood pressure causes endothelial injury, accelerated atherosclerosis, lipohyalinosis, cerebral small vessel disease, and structural cerebrovascular injury (CVI), resulting in an increased risk of stroke [4]. Several studies have shown that hypertension contributes not only to stroke occurrence but also to stroke severity, hematoma expansion, cerebral edema, and poor functional outcomes [5, 6]. Furthermore, neuroimaging markers of CVI, such as white matter hyperintensities, lacunes, cerebral microbleeds, and silent brain infarcts, have been extensively described in people with hypertension and reflect cumulative vascular injury [4, 7, 8].

With the increased westernization of lifestyles in African populations, the burden of hypertension has been rising rapidly and is projected to rise even further [2, 9]. Although older age is a risk factor for hypertension, in younger populations, the prevalence of hypertension and its associated complications has grown steadily over the last few decades [1, 10]. Yet, it remains underdiagnosed and poorly controlled, with an increased risk of cerebrovascular events at a younger age. Existing data on hypertension phenotypes and neuroimaging markers of CVI in young adults with stroke are heavily skewed towards Western populations. The phenotype of hypertension in SSA differs significantly from that of the Western population by an earlier onset, higher severity, increased salt sensitivity, and a high prevalence of low renin, nocturnal nondipping profiles [11–13]. Despite this, there remains limited data on the characteristics of hypertension and associated CVI among young adults presenting with stroke. Understanding the hypertension characteristics and the extent of CVI in young stroke patients could inform and improve risk stratification, guide prevention strategies, and support earlier intervention.

This study is aimed at describing hypertension patterns and associated CVI among young adults presenting with stroke in two tertiary hospitals in Senegal.

## 2. Methods

### 2.1. Study Design and Setting

We conducted a hospital‐based cross‐sectional study among young adults admitted with neuroimaging‐confirmed cerebrovascular events at two tertiary referral hospitals in Dakar, Senegal, from March 2023 to March 2026: the National University Hospital Centers of Fann and Pikine.

### 2.2. Study Population

#### 2.2.1. Inclusion Criteria

The population of the study are as follows:

• All patients aged ≤ 50 years with acute cerebrovascular events who underwent brain MRI with complete sequences to assess for CVI markers. The primary analyses focused on hypertension‐associated neuroimaging markers of CVI rather than stroke subtype at the study sites.
• Patients who were able to give informed consent, or informed consent was obtained from the caregiver.
• Patients who were able to communicate or obtain information on the history of hypertension from a caregiver.

#### 2.2.2. Exclusion Criteria

• Stroke mimics
• Incomplete clinical and neuroimaging data
• Missing blood pressure measurements
• Severe communication difficulties (e.g., motor or sensory aphasia)

### 2.3. Data Collection

Data were collected by the first and second authors using the Registry of Stroke quality questionnaire [14], with additional questions to collect sociodemographic and hypertension data. Additional clinical data were extracted from patients′ medical records. The following data were collected:

• Mean systolic and diastolic blood pressure at admission
• History of hypertension
• Duration of hypertension
• Compliance with treatment
• Stroke type
• NIHSS at admission
• Modified Rankin score (mRS) at discharge

Blood pressure was measured using automated sphygmomanometers. Measurements were obtained on admission after initial stabilization, with patients in the seated or supine position. Repeated measurements were performed 7 days after admission to control for acute‐phase elevations in blood pressure due to permissive hypertension and the physiological stress response, and the average value was recorded. The same standardized protocol was applied in both participating hospitals. Due to the absence of blood pressure values before admission, assessment of hypertension control could not be performed for those with a prior diagnosis of hypertension. Neuroimaging markers of CVI were assessed using brain MRI based on the Standards for Reporting Vascular Changes on Neuroimaging (STRIVE‐2) criteria [10]. Based on these criteria, neuroimaging markers were classified as lacunes, microbleeds, white matter hyperintensities (Fazekas classification), microinfarcts, brain atrophy, superficial siderosis, and perivascular space enlargement. Vascular risk factors such as diabetes, dyslipidemia, smoking, alcohol use, and cardiac disease were also assessed.

### 2.4. MRI Acquisition Protocol

MRI was performed using a standard clinical protocol for the evaluation of acute stroke and CVI. We reviewed the MRI examinations using Digital Imaging and Communications in Medicine (DICOM) images. Before image interpretation, each MRI result was assessed to ensure that the required sequences (T1‐weighted, T2‐weighted, fluid‐attenuated inversion recovery [FLAIR], diffusion‐weighted imaging [DWI] with apparent diffusion coefficient [ADC] maps, and susceptibility‐weighted imaging [SWI] or T2∗‐weighted gradient echo sequences) were present to allow a comprehensive evaluation of CVI. Consultant radiologists with training in neuroimaging and neurologists experienced in stroke imaging independently reviewed MRI scans, and neuroimaging markers of CVI were identified according to the STRIVE‐2 recommendations. Axial T1‐ and T2‐weighted and FLAIR sequences were used to assess structural brain abnormalities and white matter hyperintensities. DWI with corresponding ADC maps was used to identify acute ischemic lesions. SWI or T2‐weighted gradient echo sequences were used to detect cerebral microbleeds and hemorrhagic lesions. White matter hyperintensities were evaluated on FLAIR images and classified according to the Fazekas grading scale. MRI was acquired on a 1.5‐T system. Formal interrater reliability was not assessed.

### 2.5. Definition of Variables

A diagnosis of hypertension was made if the patient met at least one of the following criteria:

• A previously diagnosed case of hypertension, treated or untreated
• At least two average blood pressure measurements ≥ 140/90 mmHg or persistent elevation of blood pressure 7 days after stroke onset.
• A young adult with a cerebrovascular event was defined as a patient ≤ 50 years of age with a cerebrovascular event. Poor functional outcome was defined as an mRS score > 2 at discharge.
• CVI was defined as the presence of at least one neuroimaging marker of CVI, including white matter hyperintensities, lacunes, cerebral microbleeds, and silent brain infarcts, as defined by the STRIVE‐2 criteria. Acute ischemic infarcts, intracerebral hemorrhage, cerebral venous thrombosis, and other imaging abnormalities directly related to the index cerebrovascular event were not considered components of the CVI definition.
• Treatment adherence was assessed by patient self‐report at the time of hospitalization. Participants were classified as adherent if they reported consistently taking their prescribed antihypertensive medication, with occasional missed doses of no more than a day. Participants reporting more prolonged interruptions or irregular medication use were classified as nonadherent.

## 3. Statistical Analysis

The collected data were entered into an Excel spreadsheet and analyzed using SPSS Version 26. Continuous variables were expressed as mean ± standard deviation or median (interquartile range), depending on the data distribution. Categorical variables were summarized as frequencies and percentages. Comparisons between groups were performed using chi‐square or Fisher′s exact tests for categorical variables and Student′s *t*‐test or Mann–Whitney *U* test for continuous variables. A multivariable regression analysis was conducted to identify predictors of severe stroke, the presence of neuroimaging markers of CVI, and poor functional outcomes. The variables for each model were selected based on clinical relevance while considering the limited number of participants to minimize model overfitting and unstable estimates. The small number of participants who had favorable outcomes (mRS 0–2) at discharge precluded dichotomizing the mRS into categories commonly used in research. Given concerns that logistic regression based on such few events would produce unstable estimates, wide confidence intervals, and increase the risk of model overfitting, we retained mRS as a continuous outcome for the primary analysis. All *p* values < 0.05 were considered statistically significant.

## 4. Results

**Figure 1:**
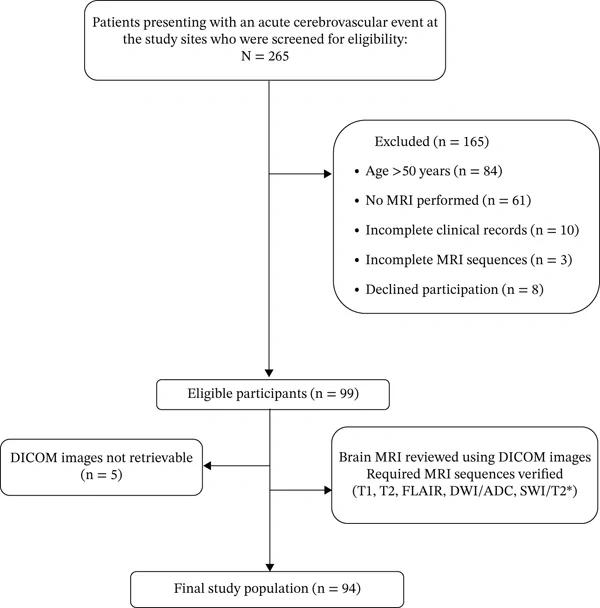
Flow diagram for the recruitment of participants.

We included 94 young adults. Figure 1 shows the recruitment flow diagram.

There was a female predominance of 55.3% (52/94) versus 44.7% (42/94). The mean age of the participants was 45.0 ± 4.3 years. Table 1 summarizes the sociodemographic characteristics of the patients.

**Table 1:** Sociodemographic characteristics of participants.

Variables	*N*	%
Sex		
Female	52	55.3
Male	42	44.7
Level of income		
< $250	59	62.7
$250–$500	28	29.8
$501–$750	3	3.2
$750–$1000	4	4.3
Marital status		
Single	27	28.7
Married	61	64.9
Divorced	5	5.3
Widow	1	1.1
Level of education		
None	10	10.6
Primary	67	71.3
Secondary	8	8.5
Tertiary	9	9.6
Employment status		
Unemployed	32	34
Employed	62	66

Most participants were married (64.9%). A sizable proportion was unemployed (34%). Among those employed, most had a monthly income of less than $250 (62.7%).

### 4.1. Hypertension Phenotypes and Treatment Characteristics

Most participants had primary hypertension, with only three cases of secondary hypertension resulting from hyperthyroidism [2] and Conn′s adenoma [1]. Table 2 summarizes the clinical characteristics of hypertension.

**Table 2:** Clinical and therapeutic characteristics of hypertension.

Variables	*N*	%
Type of hypertension		
Primary	91	96.8
Secondary	3	3.2
Hypertension status		
Previously diagnosed	56	59.6 (56/94)
Diagnosed during hospitalization for stroke	38	40.4 (38/94)
Treatment status (previously diagnosed)		
On treatment	23	41.1 (23/56)
On treatment and compliant	10	43.5 (10/23
On treatment and noncompliant	13	56.5 (13/23)
Not on treatment	33	58.9 (33/56)

### 4.2. Blood Pressures at Admission

Mean SBP at admission was 150.3 ± 31.8 mmHg (IQR: 60, 248 mmHg), whereas mean DBP was 90.83 ± 22.03 mmHg (IQR: 34, 160 mmHg). Participants who had been previously diagnosed with hypertension before admission had significantly lower SBP at admission compared to those diagnosed during admission. Mean SBP and DBP were significantly lower among participants with ischemic stroke compared to those with hemorrhagic stroke. Table 3 summarizes the mean blood pressures at admission.

**Table 3:** Blood pressures at admission.

Variable	Yes, Mean ± SD (mmHg)	No, Mean ± SD (mmHg)	Mean difference	*p*
Previously diagnosed HTN				
SBP	146.43 ± 32.07	156.03 ± 30.93	−9.60	**0.024**
DBP	89.86 ± 21.72	92.26 ± 22.71	−2.40	0.239
Treatment adherence				
SBP	149.28 ± 35.11	157.50 ± 26.24	−8.22	0.293
DBP	88.61 ± 19.32	95.60 ± 21.53	−6.99	0.287
Stroke subtype				
SBP	145.67 ± 29.69 (ischemic)	174.73 ± 32.35 (hemorrhagic)	−29.06	**0.001**
DBP	87.33 ± 19.42 (ischemic)	109.27 ± 26.26 (hemorrhagic)	−21.94	**0.001**

*Note:* Boldfaced data denote that the difference between the two groups was statistically significant.

Abbreviations: DBP, diastolic blood pressure; HTN, hypertension; SBP, systolic blood pressure.

### 4.3. Vascular Risk Profile

Left ventricular hypertrophy (27/94), atrial fibrillation (4/94), rheumatic heart disease (4/94), and Type 2 diabetes mellitus (5/94) were the most common comorbid vascular risk factors. Smoking (4/94), ischemic heart disease (4/94), infectious endocarditis (1/94), acute kidney injury (3/94), and chronic kidney disease (1/94) were infrequent. Low‐density lipoprotein (LDL) was available for 44 participants, with a mean of 1.44 ± 0.53 g/L (IQR: 0.53, 2.5).

### 4.4. Cerebrovascular Events

The most common type of cerebrovascular event was ischemic stroke. The mean NIHSS score at admission was 12.2 ± 7.53. Table 4 shows the different types of cerebrovascular events among the participants.

**Table 4:** Cerebrovascular events.

Cerebrovascular event	*N*	%
Ischemic	74	78.7
Intracerebral hematoma	13	13.8
Cerebral venous thrombosis	4	4.2
Transient ischemic attack	3	3.2
Total	94	100

### 4.5. Neuroimaging Markers of CVI

White matter hyperintensities (mean Fazekas scale score of 1.98 ± 0.69) were the most common neuroimaging marker of CVI, followed by lacunes and small subcortical infarcts. Twenty‐two (22/94) participants had two or more markers. Table 5 shows the types and frequency of neuroimaging markers of CVI.

**Table 5:** Neuroimaging markers of cerebrovascular injury.

Neuroimaging markers	*N*	%
White matter hyperintensities	45	47.9
Lacunes	17	18.1
Small subcortical infarcts	13	13.8
Brain atrophy	6	6.4
Cerebral microbleeds	3	3.2
Cortical superficial siderosis	0	0
Enlarged perivascular spaces	0	0
≥ 2 markers	22	23.4

### 4.6. Association Between Hypertension Characteristics, Stroke, and Neuroimaging Markers of CVI

Bivariate analyses were performed to assess associations between hypertension, stroke severity, neuroimaging markers, and functional outcomes. The presence of WMH was significantly associated with stroke severity and functional outcomes. Mean SBP and DBP were significantly higher in hemorrhagic strokes compared to ischemic strokes. Table 6 shows the results of the bivariate analysis.

**Table 6:** Bivariate associations between hypertension‐related variables, neuroimaging findings, stroke severity, and functional outcomes.

Association tested	*p*
WMH: stroke severity	**0.016**
Hypertension status: NIHSS	0.523
Hypertension status: CVI	0.158
Hypertension status: WMH	0.172
CVI: MRS at discharge	0.563
WMH: MRS at discharge	**0.035**
SBP at admission: NIHSS	0.711
DBP at admission: NIHSS	0.257
Stroke type: admission SBP	**0.001**
Stroke type: admission DBP	**0.001**
Duration of HTN: WMH	0.069
Duration of HTN: CVI	0.349
Hypertension status: MRS	0.345
SBP at admission: MRS	0.877
DBP at admission: MRS	0.347
CVI: NIHSS	**0.014**
CVI: LDL	0.089 g/L

*Note:* Values are presented as mean ± standard deviation (SD); *p* values were obtained using the Mann–Whitney test. Boldfaced data denote that the association between the two variables was statistically significant.

Abbreviations: CVI, cerebrovascular injury; DBP, diastolic blood pressure; g/L, grams per liter; LDL, low‐density lipoprotein; mRS, modified Rankin Scale; NIHSS, National Institutes of Health Stroke Scale; SBP, systolic blood pressure; WMH, white matter hyperintensities.

Participants with hypertension duration ≥ 5 years had 4.94 times higher odds of WMH compared with those with shorter hypertension duration, but this was not statistically significant (**p** = 0.069, CI: 0.54–45.11).

A multivariable linear regression analysis was performed to identify predictors of NIHSS score at admission. The overall model was not statistically significant (*F* = 1.71, *p* = 0.127). However, after adjusting for age, sex, prior hypertension diagnosis, stroke type, SBP, and DBP at admission, the presence of a neuroimaging marker of CVI remained significantly associated with higher admission NIHSS scores (*B* = 3.35, 95% CI 0.18–6.49, *p* = 0.032). Table 7 shows the results of the multivariable regression analysis.

**Table 7:** Multivariable linear regression analysis of predictors of NIHSS score at admission.

	*p*	95% CI
Age	0.483	−0.66, 0.32
Sex male	0.18	−5.47, 1.04
Previously diagnosed: yes	0.464	−2.02, 4.39
SBP at admission	0.511	−0.10, 0.05
DBP admission	0.144	−0.03, 0.19
Stroke type: hemorrhagic	0.935	−4.43, 4.81
CVI present: yes	**0.032**	0.18, 6.49

*Note:* Boldfaced data denote that the association between the two variables was statistically significant

Abbreviations: CVI, cerebrovascular injury; DBP, diastolic blood pressure; SBP, systolic blood pressure.

No significant predictors of functional outcome at discharge were identified in multivariable analysis, and the overall regression model was not statistically significant (**F** = 0.39, **p** = 0.902). Table 8 shows the results of the multivariable regression analysis.

**Table 8:** Multivariable linear regression analysis of predictors of mRS at discharge.

	*p*	95% CI
Age	0.795	−0.16, 0.20
Sex male	0.973	−0.93, 0.96
Previously diagnosed: yes	0.319	−0.47, 1.42
SBP at admission	0.664	−0.02, 0.03
DBP admission	0.953	−0.05, 0.04
Stroke type: hemorrhagic	0.39	−2.02, 0.80
CVI present: yes	0.298	−0.46, 1.46

Abbreviations: CVI, cerebrovascular injury; DBP, diastolic blood pressure; SBP, systolic blood pressure.

Multivariable logistic regression analysis was performed to identify predictors of CVI. No independent predictors of CVI were identified. Table 9 shows the results of the multivariable regression analysis.

**Table 9:** Multivariable logistic regression analysis of predictors of CVI.

	*p*	Odds ratio	95% CI
Age	0.88	0.99	0.81, 1.20
Sex: male	0.367	0.59	0.19, 1.86
Duration of HTN > 5	0.16	2.89	0.66, 12.66
Stroke type: hemorrhagic	0.295	2.35	0.47, 11.69

Abbreviation: HTN, hypertension.

A sensitivity analysis was performed after excluding participants with cerebral venous thrombosis (**n** = 4) and transient ischemic attack (**n** = 3), leaving 87 participants with ischemic or intracerebral hemorrhagic stroke. White matter hyperintensities remained significantly associated with stroke severity and functional outcome, while there were no independent predictors of CVI identified in multivariable analyses.

## 5. Discussion

This study aimed to describe hypertension characteristics, stroke severity, and neuroimaging markers of CVI and to assess the relationship between them among young adults presenting with cerebrovascular events. A substantial proportion of participants had newly dia hypertension, whereas among those previously known to have hypertension, a sizable proportion were not on treatment. Also, among those on treatment, only a small proportion were compliant. This pattern of undiagnosed and poorly treated hypertension is a well‐documented public health challenge, particularly among younger demographics in sub‐Saharan Africa [1, 2, 11, 13]. This could be due to critical gaps in healthcare delivery and to patients′ perceptions of risk [2]. Because hypertension is largely asymptomatic in the early stages, young adults may wrongly assume that they are in good health and may not routinely go for screening. Even when diagnosed, compliance is significantly low in younger patients with hypertension. It may be due to barriers such as lack of perceived urgency, pill burden, and lifestyle, as well as socioeconomic factors such as financial constraints [11, 15, 16]. This echoes the need to reinforce preventive cardiovascular care for young adults in SSA through routine population screening, community education, and simplified therapeutic regimens to promote adherence [17, 18].

Mean admission SBP and DBP of participants who had been previously diagnosed were lower than those of participants who had not been previously diagnosed. Similarly, participants who were compliant with treatment had lower mean SBP and DBP than those who were not. These could be explained by the effect of ongoing antihypertensive medication use in the previously diagnosed group, as well as the treatment‐compliant group, and the fact that participants in these groups might have also adopted some healthy lifestyle changes after being diagnosed [19]. Although these differences did not translate to less severe strokes or better outcomes, probably due to the small sample size and challenges with stroke care in these settings, they reinforce the importance of antihypertensive treatment for blood pressure lowering.

Similar to previous results from the literature [6, 20–23], mean SBP and DBP were significantly higher for hemorrhagic stroke compared to ischemic stroke. In hemorrhagic stroke, the mass effect from blood accumulation can lead to elevated intracranial pressure and reduced cerebral perfusion pressure. In response, the body raises systemic blood pressure to preserve cerebral blood flow [6, 24]. Furthermore, hemorrhage can trigger catecholamine release, a sympathetic surge, and hypothalamic stress pathways. These can result in vasoconstriction, tachycardia, and markedly elevated blood pressures. Further, patients with hemorrhagic stroke are often agitated and in pain, factors that can raise blood pressure. This finding is consistent with the established role of severe hypertension in the pathogenesis of intracerebral hemorrhage.

A high burden of CVI, as evidenced by neuroimaging markers, was observed in this cohort, with a predominance of white matter hyperintensities. WMH is the most commonly reported neuroimaging manifestation of cerebral small vessel disease and results from chronic ischemic injury to deep white matter tracts [7, 8, 25–27]. The underlying mechanism involves chronic hypertension, which contributes to endothelial dysfunction, lipohyalinosis, arteriolar wall thickening, impaired autoregulation, and disruption of the blood–brain barrier [27]. Over time, these processes result in diffuse white matter injury, reduced cerebral perfusion reserve, and increased vulnerability to acute cerebrovascular insults. These findings suggest that neuroimaging evidence of chronic vascular brain injury may better reflect the cumulative burden of hypertension‐mediated cerebrovascular damage than hypertension history alone.

One of the key findings of this study was the significant association between WMH and both stroke severity and functional outcomes. Participants with WMH demonstrated more severe neurological deficits at presentation and greater poststroke disability at discharge. These findings align with prior neuroimaging studies demonstrating that WMH are associated with increased stroke severity, cognitive decline, impaired recovery, recurrent stroke, and long‐term disability [25, 27–30]. Chronic white matter injury may reduce cerebral resilience and impair compensatory collateral circulation during acute ischemic or hemorrhagic events [6, 27]. Patients with extensive small vessel disease may therefore have diminished capacity to tolerate additional vascular insults, resulting in more severe neurological deficits. Additionally, WMH may reflect widespread microvascular dysfunction that extends beyond the visible lesions identified on MRI or CT. Such diffuse vascular pathology may contribute to impaired neurovascular coupling, chronic hypoperfusion, and reduced neural network integrity, thereby amplifying the neurological consequences of acute stroke [8]. However, the significant association between WMH and stroke severity and outcome in our study should be interpreted cautiously as the cross‐sectional design limits causal interpretation.

The association of WMH with worse functional outcomes at discharge confirms the prognostic value of chronic CVI in stroke and may be explained by several interacting mechanisms. White matter integrity is essential for efficient communication between cortical and subcortical brain regions that are critical for motor recovery [31, 32]. White matter injury may impair motor recovery, cognitive processing, executive function, and adaptive neuroplasticity following stroke [32]. Furthermore, WMH‐induced reductions in brain reserve may limit the capacity for functional compensation after acute neurological injury. Previous studies have similarly shown that patients with greater WMH burden experience increased disability, delayed rehabilitation, recurrent vascular events, and reduced long‐term independence [28, 30]. Overall, these findings suggest that neuroimaging markers of chronic vascular injury may provide important prognostic information beyond clinical variables, as reported in similar studies across diverse populations [30].

Although cerebral small vessel disease is usually more prevalent among older populations, its presence in relatively young adults could result in accelerated cerebrovascular aging and early development of cognitive decline.

The absence of a significant association between previously diagnosed hypertension and CVI may be explained by several factors. First, previously diagnosed hypertension may not accurately reflect cumulative blood pressure burden over time. Many patients without a formal diagnosis may still have experienced prolonged periods of undetected hypertension and progressive vascular injury. Second, antihypertensive treatment among previously diagnosed patients may have partially mitigated ongoing cerebrovascular damage. Third, the modest sample size may have limited statistical power to detect independent associations after multivariable adjustment.

Although several univariable associations were identified in the bivariate analyses, multivariable regression analyses did not identify independent clinical predictors of CVI or functional outcome. CVI remained independently associated with higher NIHSS scores at admission. However, the relatively wide confidence interval indicates uncertainty around the magnitude of the association. Several methodological and statistical factors likely contributed to these negative multivariable findings; the modest sample size may have limited statistical power, especially given the number of variables included in the regression models. Also, CVI represented a heterogeneous outcome incorporating multiple imaging abnormalities with potentially distinct pathophysiological mechanisms. Consequently, standard regression techniques may fail to generate stable independent estimates despite the presence of clinically significant relationships. Therefore, the absence of statistically significant independent predictors should not be interpreted as evidence against the importance of hypertension‐mediated CVI in young adults. Further studies with larger sample sizes should address these methodological issues.

### 5.1. Clinical Implications

Firstly, although older age is a well‐described risk factor of CVI, our results suggest vulnerability to hypertension‐mediated CVI among young adults. Secondly, neuroimaging markers such as WMH could be incorporated into risk stratification frameworks for young stroke patients. Furthermore, the high burden of neuroimaging markers of CVI observed in this cohort echoes the urgent need to promote primary prevention, routine hypertension screening, improved treatment adherence, and aggressive vascular risk reduction strategies. Finally, the findings support evidence that chronic CVI contributes not only to stroke occurrence but also to stroke severity and functional outcomes.

### 5.2. Strengths and Limitations of the Study

The use of MRI markers increased the accuracy of characterizing cerebrovascular damage. Additionally, we evaluated multiple neuroimaging markers and characterized hypertensive and blood pressure profiles among a relatively young population that remains understudied in Africa. To the best of our knowledge, this is one of the few studies describing CVI using MRI markers in African populations. The focus on neuroimaging markers of CVI further strengthens the mechanistic and translational relevance of the findings. However, the cross‐sectional design precludes causal inference. Also, the overall sample size and the sample size available for some analyses were relatively modest. These might have substantially reduced the study′s statistical power. Fourth, neuroimaging protocols may not have been completely standardized across all participants. Importantly, the contributions of other vascular risk factors, such as adverse lifestyle factors, diabetes mellitus, obesity, atrial fibrillation, and dyslipidemia, were not assessed because of the small number of participants with these factors in our cohort. The total risk of CVI, stroke severity, and functional outcomes rises with the cumulative burden of these vascular risk factors. Blood pressure measurements were taken in the hospital using automated devices rather than the recommended longitudinal approach of ambulatory blood pressure monitoring. These could have affected the accuracy of the blood pressure values in our study. Blood pressure was measured during the acute phase of stroke and may be influenced by the physiological stress response; therefore, it may not fully reflect baseline blood pressure levels. Multiple exploratory bivariate analyses were performed without formal adjustment for multiple comparisons. Consequently, statistically significant findings should be interpreted cautiously and considered hypothesis‐generating until confirmed in larger prospective studies. Treatment adherence was assessed by patient self‐report rather than by objective measures such as pharmacy refill records or pill counts. Consequently, recall bias and social desirability bias may have resulted in some misclassification of adherence status. Finally, certain etiologies of ischemic stroke may strongly influence the occurrence of CVI. Although we collected data on TOAST classifications for participants, more than 70% of ischemic strokes were classified as undetermined etiology because of incomplete investigations. The relatively small number of participants in the remaining TOAST subgroups precluded meaningful subgroup analyses examining the association between hypertension‐related CVI and individual ischemic stroke mechanisms.

## 6. Conclusion

These findings suggest that chronic CVI may be an important contributor to stroke severity and functional outcomes young adults with cerebrovascular events in this setting. A substantial burden of hypertension‐related CVI, severe neurological deficits, and significant poststroke disability was observed among young adults. Neuroimaging markers of chronic CVI, particularly WMH, were more associated with stroke severity and functional outcomes than hypertension history alone. Large‐scale prospective studies are needed to validate these findings.

## Funding

No funding was received for this manuscript.

## Ethics Statement

This study was part of a larger study on the burden of stroke in Senegal, which received ethical approval from the National University Teaching Hospital of Pikine (No. 000704/MSHP/DGES/CHNP/DIR/SRH). The study was conducted in accordance with the principles of the Declaration of Helsinki.

## Consent

Informed patient consent was obtained from all patients. Participants had the right to withdraw at any time.

## Conflicts of Interest

The authors declare no conflicts of interest.

## Data Availability

The data that support the findings of this study are available on request from the corresponding author. The data are not publicly available due to privacy or ethical restrictions.
